# Spatiotemporal dynamics of the 2025–26 measles resurgence in Mexico: A national scan‑statistic analysis

**DOI:** 10.1371/journal.pone.0353018

**Published:** 2026-07-06

**Authors:** Oliver Mendoza-Cano, Xóchitl Trujillo, Mónica Ríos-Silva, Jaime Alberto Bricio-Barrios, Agustin Lugo-Radillo, Rosa Tapia-Vargas, Jesús Venegas-Ramírez, Eder Fernando Ríos-Bracamontes, Herguin Benjamin Cuevas-Arellano, Yolitzy Cárdenas, Raúl Aquino-Santos, Iris Anecxi Jiménez-Vieyra, Juan Manuel Uribe-Ramos, Verónica Benites-Godínez, Teresa Evangelina Martínez-Díaz, Mario López-Rojas, Annel García-Solórzano, José E. Velázquez-Amador, Efrén Murillo-Zamora

**Affiliations:** 1 Facultad de Ingeniería Civil, Universidad de Colima, Coquimatlán, Mexico; 2 Centro Universitario de Investigaciones Biomédicas, Universidad de Colima, Colima, Mexico; 3 Facultad de Medicina, Universidad de Colima, Colima, Mexico; 4 SECIHTI - Facultad de Medicina y Cirugía, Universidad Autónoma Benito Juárez de Oaxaca, Oaxaca, Mexico; 5 Coordinación de Planeación y Enlace Institucional, Jefatura de Servicios de Prestaciones de Médicas, Instituto Mexicano del Seguro Social, Colima, Mexico; 6 Coordinación Auxiliar Médica de Investigación en Salud, Jefatura de Servicios de Prestaciones Médicas, Instituto Mexicano del Seguro Social, Colima, Mexico; 7 Departamento de Medicina Interna, Hospital General de Zona No. 1, Instituto Mexicano del Seguro Social, Villa de Álvarez, Mexico; 8 Facultad de Ciencias, Universidad de Colima, Colima, Mexico; 9 Coordinación General de Investigación, Universidad de Colima, Colima, Mexico; 10 Coordinación de Educación en Salud, Jefatura de Servicios de Prestaciones Médicas, Instituto Mexicano del Seguro Social, Tepic, Mexico; 11 Unidad Académica de Medicina Humana, Universidad Autónoma de Nayarit, Tepic, Mexico; 12 Coordinación Auxiliar Médica de Educación en Salud, Jefatura de Servicios de Prestaciones Médicas, Instituto Mexicano del Seguro Social, Colima, Mexico; 13 Universidad Autónoma de Guadalajara, Facultad de Medicina, Zapopan, Mexico; 14 Unidad de Investigación en Epidemiología Clínica, Instituto Mexicano del Seguro Social, Villa de Álvarez, Mexico; Universidad Cooperativa de Colombia, COLOMBIA

## Abstract

**Background:**

Following over two decades of eliminated endemic transmission, Mexico experienced a significant measles resurgence in 2025–26. Declining vaccine coverage and pandemic-related service disruptions created subnational immunity gaps. We aimed to characterize the spatiotemporal patterns of this outbreak to identify areas of sustained transmission.

**Methods:**

We conducted a retrospective surveillance study of all confirmed measles cases reported nationally over a 50-week period (mid-February 2025 to February 10, 2026). Using municipal-level data and population projections, we applied Kulldorff’s space–time scan statistic (discrete Poisson model) to identify statistically significant clusters where incidence exceeded population-based expectations.

**Results:**

A total of 8,205 confirmed cases were reported across 337 municipalities during the study period. Overall, 23 space–time clusters were identified, of which 19 were statistically significant (*p* < 0.001). The primary cluster, located in northern Mexico between epidemiological weeks 6 and 30, represented the focus of transmission, encompassing 89 municipalities and 4,012 cases (relative risk [RR] = 103.7). Secondary clusters reflected additional transmission patterns, including a large late-phase cluster in western Mexico with 1,493 cases (RR = 31.5), as well as several smaller, short-duration micro-outbreaks with markedly elevated localized risk (RR > 200).

**Conclusions:**

The resurgence of measles was characterized by marked subnational heterogeneity, with both sustained regional transmission and localized surges. A late-stage, low-intensity cluster spanning 244 municipalities suggested a transition toward more diffuse national transmission. These spatiotemporal patterns suggest that geographically differentiated approaches, including subnational surveillance and targeted “mop-up” immunization campaigns, may be needed to address immunity gaps and support efforts toward restoring Mexico’s elimination status.

## Introduction

Characterized by an unparalleled transmission potential, measles remains one of the most contagious human pathogens known to medicine, with a basic reproduction number (*R*_0_) frequently exceeding 12–18 [1]. Although the Americas achieved regional elimination status in 2016 [2], recurrent importations, declining vaccination coverage, and persistent subnational heterogeneities have created critical immunity gaps, undermining regional progress [3].

Several countries in the region have experienced substantial resurgences since 2017. Venezuela and Brazil reported large outbreaks beginning in 2017–2018, leading to re-establishment of endemic transmission and temporary loss of measles elimination status. Venezuela reported 7,054 confirmed cases and 84 deaths between 2017 and 2019 [4], while Brazil reported over 21,700 cases in 2019 alone [5]. In the United States, 1,249 measles cases were confirmed in 2019, the highest number since 1992, with 75% linked to outbreaks in New York among undervaccinated communities [6]. Between 2018 and 2023, the Americas reported 49,187 confirmed cases across 18 countries [7]. These resurgences reflect a broader global trend in which health system disruptions [8] and population mobility have eroded the resilience of routine immunization programs.

Mexico sustained interruption of endemic measles transmission for over two decades. Surveillance data indicate that indigenous virus circulation ceased in 1997, with zero confirmed cases during 1997–1999 [9]. Following this period, the country reported only sporadic, import-related outbreaks (30 laboratory-confirmed cases in 2000 linked to a Canadian visitor) [9], and 176 cases during an importation-driven outbreak in early 2020 (February–June), concentrated in Mexico City and adjacent municipalities [10,11]. Both outbreaks were rapidly contained, and no sustained endemic transmission was documented. However, national vaccination coverage declined steadily from 2013 onward, dropping to 79% in 2017 and ranging from 83–89% (average 87%) during 2013–2018 [11].

Mexico’s national immunization program administers measles-containing vaccine (MCV) as the combined measles-rubella-mumps (SRP) vaccine at 12 months (first dose) and 6 years of age (second dose). First-dose MCV coverage declined from 88% in 2019 to 84% in 2020, recovering to 91% by 2023, with persistent subnational heterogeneity and several states consistently below the 95% herd immunity threshold [12]. These vulnerabilities were compounded by pandemic-related disruptions [13]. Serological surveys in 2022 revealed critically low measles-neutralizing antibody prevalence among young adults aged 20–29 years (63.6%), indicating accumulated immunity gaps [14].

The 2025–26 measles resurgence has not been systematically characterized in its spatial and temporal structure. To date, no spatiotemporal analysis using prospective scan statistics has been published for this outbreak. Understanding where and when transmission was most intense, whether driven by sustained regional amplification, repeated introductions, or short-duration micro-outbreaks, is essential for designing geographically targeted vaccination strategies and strengthening subnational surveillance [15–17].

Traditional descriptive epidemiology has limitations in detecting statistically significant clusters, quantifying excess risk, and delineating the precise geographic footprint of transmission [18]. Space–time scan statistics offer a robust framework for identifying localized excess incidence and characterizing transmission dynamics across heterogeneous settings [19]. Given Mexico’s large geographic size, federal structure, demographic diversity, and documented heterogeneity in immunization coverage and health-system performance, this approach can reveal complex subnational patterns that are not apparent from national aggregates, and enables public health authorities to prioritize resource allocation, deploy geographically targeted vaccination campaigns, and strengthen surveillance in areas with sustained transmission.

This study leverages national measles surveillance data to characterize the spatial and temporal dynamics of the 2025–26 outbreak in Mexico. Our objective was to identify and describe statistically significant space–time clusters of measles incidence, quantify their magnitude, duration, and geographic extent, and assess their contribution to the national outbreak trajectory.

## Methods

### Study design

We conducted a retrospective observational study of measles transmission dynamics in Mexico using national surveillance data from the General Directorate of Epidemiology (DGE) [20]. The dataset was retrieved on Feb 16, 2026, from the official open‑data repository and included all confirmed cases with a diagnosis date up to Feb 10, 2026. Available variables comprised demographic characteristics (age and sex), geographic identifiers (municipality and state of residence), final case classification, and diagnostic criteria (clinical, epidemiological, or laboratory-based). Diagnosis date was used as a proxy for symptom onset due to inconsistent reporting of the latter in the public database.

A disruption in data availability occurred between late 2025 and early 2026. Although the dataset released on Dec 17, 2025, remained accessible, a subsequent update dated Dec 26, 2025, could not be retrieved due to persistent server errors (HTTP 404). This resulted in a reporting gap from Dec 16, 2025, to Jan 4, 2026. The discontinuity was explicitly addressed during temporal preprocessing to minimize bias in the spatiotemporal analysis.

### Setting

Mexico comprises 32 federal entities (31 states and Mexico City), in turn subdivided into 2,471 municipalities, the smallest administrative units for which population and health surveillance data are systematically collected. The 2025 national population was estimated at 129 million inhabitants, with substantial heterogeneity in population density, urbanization, and access to health services across federal entities. This study covered the entire national territory, with the municipality as the geographic unit of analysis for spatiotemporal scan statistics. All confirmed measles cases and population denominators were aggregated at the municipal level. A map illustrating the geographical division of Mexico’s federal entities is provided as S1 Fig, and a reference map of the same boundaries is also available from the National Institute of Statistics and Geography (INEGI) [21].

### Case definition and population data

Measles case definitions followed Mexico’s official epidemiological surveillance guidelines for vaccine-preventable diseases [22]. A probable case was defined as any person presenting with fever, maculopapular rash, and one or more of the following: cough, coryza, conjunctivitis, or lymphadenopathy (retroauricular, occipital, or cervical). A confirmed case was defined as any probable case with laboratory-confirmed measles virus infection through IgM antibodies or RT-PCR or epidemiologically linked to a laboratory-confirmed case. A discarded case was a probable case with negative laboratory results or ruled out by expert committee review. The “1” diagnostic code in the surveillance database corresponds to confirmed cases.

The initial dataset included 21,104 suspected cases (14,430 in 2025; 6,674 in 2026), of which 8,205 were confirmed (5,738 in 2025; 2,467 in 2026). Laboratory confirmation was available for 94.4% of confirmed cases (7,745/8,205), with the remainder classified through clinical and epidemiological association.

Population denominators were obtained from the National Population Council (CONAPO) 2025–26 municipal projections, calculated as the sum of male and female estimates [23]. Incidence rates were expressed per 100,000 inhabitants and classified into discrete categories (“No cases”, 1–10, 11–50, 51–150, 151–400, and > 400 per 100,000) to improve interpretability given the highly skewed distribution of municipal rates.

### Data preparation and geospatial analysis

Geographic identifiers were standardized into five-digit municipality codes by concatenating state (01–32) and municipality (three-digit) identifiers, consistent with the INEGI framework. A vector layer was constructed using the 2025 Geostatistical Framework [24], and municipal centroids were calculated to serve as the coordinate base for spatial analysis.

Data were aggregated into a Case-Aggregation Structure (CAS) by municipality and epidemiological week. Cases were assigned to epidemiological weeks based on diagnosis date. The study period spanned 50 epidemiological weeks from February 19, 2025 (Epidemiological Week 8) to February 10, 2026. Weekly case counts at the municipal level served as the input for spatiotemporal scan statistics. Weekly incidence rates were calculated for each municipality as the number of confirmed cases per 100,000 inhabitants per week, using CONAPO’s 2025 mid-year population estimates as fixed denominators. Cumulative incidence for the entire study period was calculated as a summary measure (total cases per 100,000 inhabitants) for descriptive purposes only and was not used in spatiotemporal analysis.

To maintain the continuous temporal sequence required for scan statistics despite the mid-December reporting gap, a temporal realignment was performed. Week 1 was defined as the week of the first confirmed case (Feb 19, 2025; Epidemiological Week 8), and subsequent weeks were numbered sequentially; this renumbering was purely cosmetic for intuitive presentation and had no impact on the spatiotemporal analysis, as SaTScan operates on actual dates rather than numerical week labels.

The period with missing data (previously Weeks 45–46) was bypassed by recoding the next active week (Week 47) as the subsequent chronological step (Week 45), preserving temporal contiguity without introducing artificial zero-case intervals. Three alternatives were considered for handling this gap: i) inserting artificial zero-case weeks, which would violate Poisson model assumptions and introduce spurious temporal patterns; ii) truncating the analysis before the gap, discarding valuable late-outbreak-phase information; or iii) removing the gap period while preserving temporal continuity. We selected third option as the most conservative approach, maintaining correct temporal sequence for scan statistics while acknowledging potential underestimation during that interval.

### Spatial analysis and mapping

Spatial analyses and mapping were conducted in R 4.4.1 (R Core Team) using the ggplot2 package. The municipal shapefile used as the base map was developed by the research team from publicly accessible geographic coordinates of municipal boundaries provided by INEGI’s 2024 Geostatistical Framework. No proprietary basemaps, tiles, or satellite imagery were used. All spatial layers were projected to the official national grid (EPSG: 6362) for consistency across analyses.

### Spatiotemporal statistical analysis

Spatiotemporal clusters of measles incidence were identified using Kulldorff’s space–time scan statistic, implemented in SaTScan version 10.3.3 (Martin Kulldorff and Information Management Services Inc.; Boston, MA, USA). A discrete Poisson model was used, if the number of cases in each municipality followed a Poisson distribution proportional to the population at risk. The scanning window was defined as a cylinder with a circular geographic base and a temporal height. The maximum spatial window size was set to 50% of the population at risk, and the maximum temporal window to 50% of the study period. These thresholds represent standard recommendations in scan statistic methodology, balancing sensitivity to detect large-scale regional transmission with specificity to avoid overly broad clusters lacking actionable geographic precision. Purely spatial and purely temporal clusters were not evaluated, as our research objective required identifying where and when transmission was most intense simultaneously to inform both geographic targeting and response timing. Statistical significance was assessed using 999 Monte Carlo replications, and clusters were reported under the no-geographical-overlap criterion.

Single-municipality clusters were retained if they met the statistical significance threshold (p < 0.001) and the minimum case requirement (≥2 cases). The potential for false-positive detection was addressed through the 999 Monte Carlo replications (correcting for multiple testing), the stringent significance threshold, and the discrete Poisson model which accounts for expected random variation based on population size and temporal distribution. Single-municipality clusters with markedly elevated relative risk represent epidemiologically plausible microoutbreaks, consistent with measles transmission dynamics following introduction into highly susceptible populations.

All data preprocessing and visualization were conducted in R version 4.5.2 (R Foundation for Statistical Computing; Vienna, Austria) using the tidyverse [25], sf [26], lubridate [27], and data.table [28] packages. The full SaTScan parameter file, along with the case (CAS), population (POP), and geographic coordinates (GEO) input files, are provided as open data to ensure full reproducibility.

### Ethical considerations

This study used de-identified, aggregated public health data from open-access repositories. As the data were anonymized and available in the public domain, the study was exempt from review by an Institutional Review Board, and informed consent was not required.

## Results

### Overview of national measles activity

A total of 8,205 confirmed measles cases were included in the spatiotemporal analysis, distributed across 337 of Mexico’s 2,471 municipalities (13.7%) during the 50‑week study period. Among all confirmed cases, 50.7% were female (*n* = 4,164) and 49.3% male (*n* = 4,044). The median age was 17 years (interquartile range 5–29), with a broad age distribution: 9.6% were infants < 1 year, 14.6% aged 1–4 years, 21.1% aged 5–14 years, 29.7% aged 15–29 years, 22.5% aged 30–49 years, and 2.5% aged ≥ 50 years.

The mean weekly incidence rate was 50.0 cases per 100,000 population (SD 142.7), with a median of 5.4 (IQR 1.5–23.6). The highest municipal rates occurred in four municipalities in Chihuahua (1,122.1 [week 32], 1,079.2 [week 19], 790.0 [week 4], and 746.6 [week 17]) and in one municipality in Guerrero (745.6, week 44).

**Fig 1:**
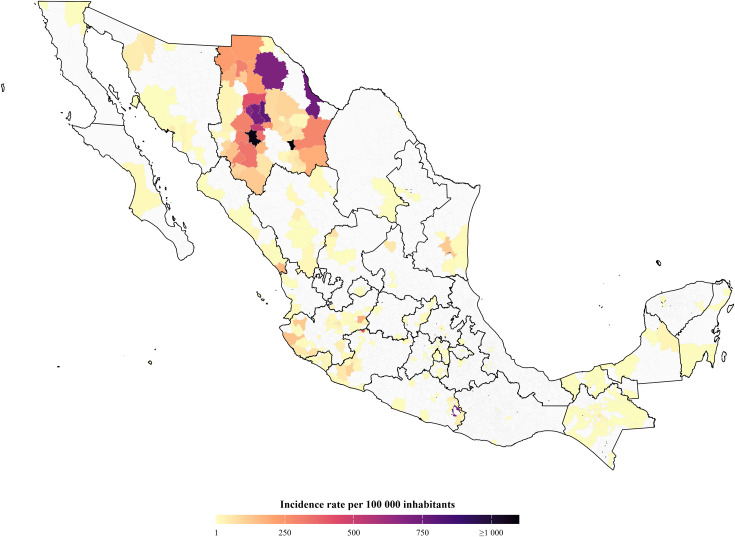
Cumulative measles incidence rate, Mexico, 2025–26. Notes: Municipalities shown in grey reported no confirmed measles cases during the 50‑week study period. The map uses the Lambert Conformal Conic coordinate system (EPSG:6362).

Fig 1 shows cumulative measles incidence rates per 100,000 inhabitants using a discrete, categorized color scale designed to improve interpretability given the highly skewed distribution of municipal rates. Municipalities were grouped into six incidence intervals (“No cases”, 1–10, 11–50, 51–150, 151–400, and > 400 per 100,000), allowing clearer visual comparison across regions. This representation highlights a highly focal and heterogeneous spatial pattern: the highest incidence was concentrated in selected municipalities of Chihuahua, where small populations and clustered cases produced rates in the 750–1,100 per 100,000 range. Additional high‑incidence pockets were observed in parts of central and western Mexico, whereas most municipalities fell within the lowest categories or reported no cases, indicating geographically circumscribed outbreaks against a backdrop of widespread apparent absence of transmission.

### Spatial heterogeneity and cluster detection

**Fig 2:**
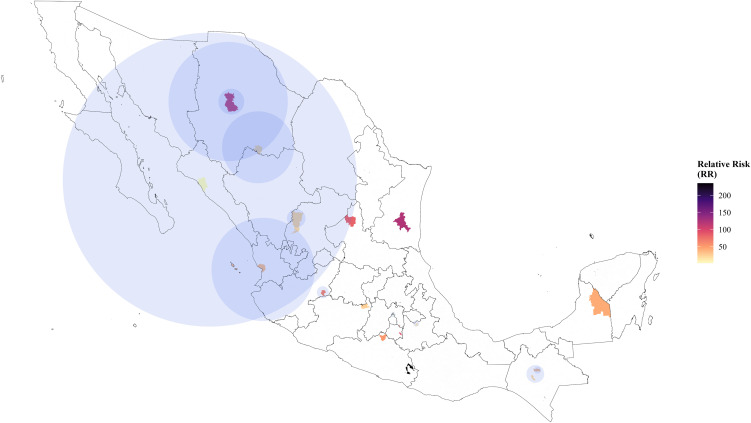
Independent SaTScan-detected space–time clusters of elevated measles risk and municipal relative risk (RR) in Mexico, 2025–26. Notes: Only the seven principal clusters are shown. The map uses the Lambert Conformal Conic coordinate system (EPSG:6362).

Building on this spatial heterogeneity, the space–time scan statistic identified 23 clusters of elevated risk, of which 19 were statistically significant, confirming that the observed hotspots represent true excess transmission rather than random variation. These clusters were highly concentrated in a limited number of municipalities, consistent with the localized patterns shown in Fig 2.

### Primary cluster

The most likely cluster was in northern Mexico, with a centroid at 28.72°N, 106.95°W and a spatial radius of 295 km, encompassing 89 municipalities. SaTScan identified an optimal temporal window spanning epidemiological weeks 6–30. Within this cluster, 4,012 cases were reported compared with 75.0 expected, yielding an observed-to-expected ratio of 53.5 and a relative risk (RR) of 103.7. The aggregated incidence was 2,403.6 per 100,000 population. The cluster had a log-likelihood ratio of 13,187.8 and a p value < 10 ⁻ ¹⁷, indicating a highly significant concentration of transmission in space and time.

**Fig 3:**
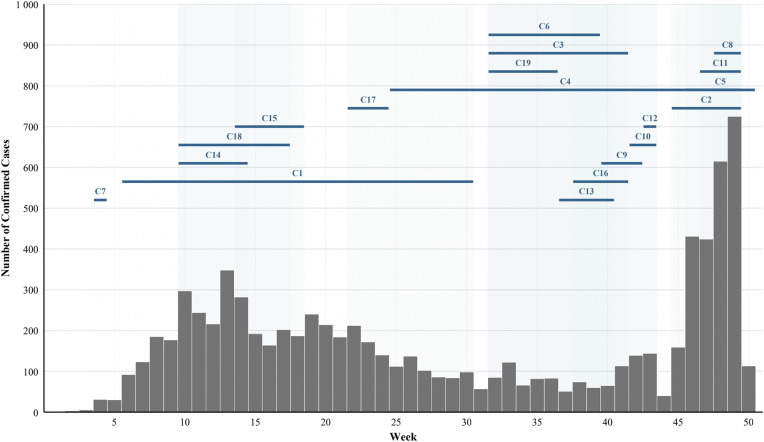
Weekly number of confirmed measles cases during the 50‑week study period, with the temporal extent of all detected space–time clusters, Mexico, 2025–26. Notes: C labels denote cluster identifiers. A disruption in data availability between mid‑December 2025 and early January 2026 created a reporting gap, which was corrected through temporal realignment to maintain a continuous weekly sequence for the spatiotemporal analysis.

Fig 3 places this cluster within the national epidemic curve by overlaying its temporal window onto the weekly number of confirmed cases. The rise in incidence during weeks 6–27 overlaps with the active period of the primary cluster. The figure also shows that subsequent fluctuations in weekly case counts occur during the time windows of several secondary clusters.

**Fig 4:**
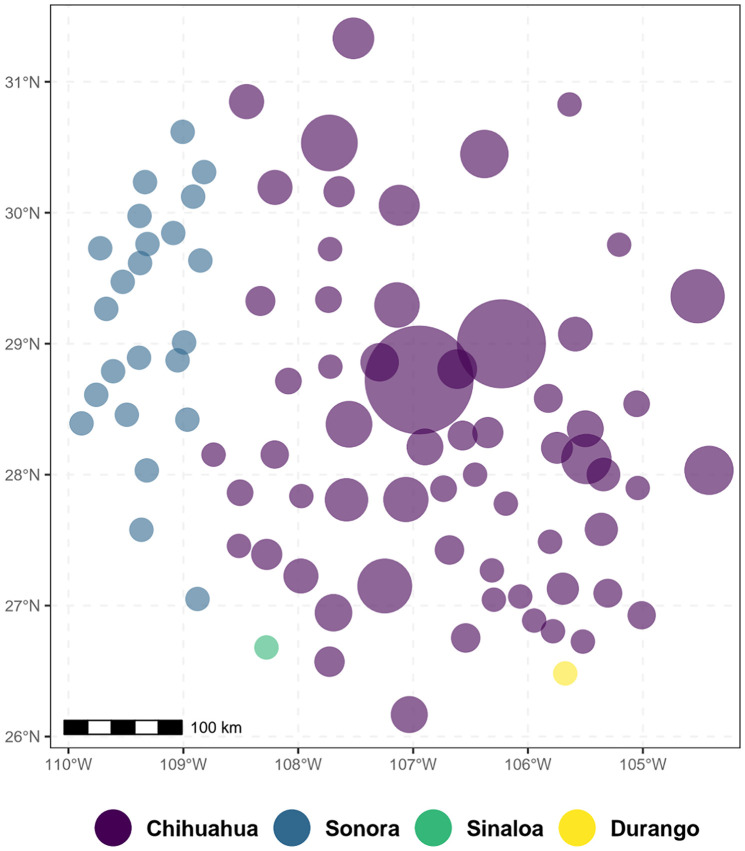
Spatial distribution and case burden of municipalities within the primary measles cluster, Mexico, 2025–26. Notes: Map of northern Mexico showing the 89 municipalities included in the primary space–time cluster detected by SaTScan (centroid 28.72°N, 106.95°W; radius 295 km) during epidemiological weeks 6–30. Relative risk is shown as a continuous background surface, while bubble size is proportional to the number of confirmed cases in each municipality. The cluster spans central and western Chihuahua and extends into adjacent areas of Sonora, Durango, and Sinaloa.

The spatial extent of this high-intensity aggregation is shown in Fig 4, which maps the 89 municipalities encompassed by the primary cluster across northern Mexico. The figure illustrates the broad, contiguous zone of high risk spanning central and western Chihuahua and extending into adjacent areas of Sonora, Durango, and Sinaloa. The large radius of 295 km reflects the wide geographic reach of sustained transmission during weeks 6–30, consistent with the high observed-to-expected ratio and relative risk.

### Secondary clusters

Secondary clusters were detected in multiple regions of the country. The largest (cluster 2) occurred in western Mexico, centred at 21.57°N, 105.49°W with a radius of 272 km, spanning weeks 45–49 and comprising 113 municipalities. This cluster contained 1,493 observed cases versus 57.5 expected (RR 31.5), corresponding to an aggregated incidence of 1 166·8 per 100 000 population.

A third high-intensity cluster (cluster 3) was detected in the Bajío region, centred at 20.49°N, 102.34°W with a radius of 31 km, spanning weeks 32–41. It included 295 cases versus 4.7 expected (RR 64.8), reflecting a high observed‑to‑expected ratio within a geographically limited area.

Several smaller clusters showed extreme localized risk. A single-municipality cluster (cluster 4) in southern Mexico persisted from weeks 25–49 and reported 134 cases versus 0.58 expected (RR 235.8). Additional single-location clusters showed similarly elevated risks, including cluster 9 (RR 54.2), cluster 15 (RR 24.5), and cluster 16 (RR 92.1).

Other clusters were more diffuse but still significant. Cluster 5 in southeastern Mexico spanned weeks 46–50, comprising 22 municipalities and reporting 193 cases versus 9·3 expected (RR 21.2). A mid-outbreak cluster in north-central Mexico (cluster 6) spanned weeks 32–39, with 108 cases versus 7.2 expected (RR 15.1). Several additional clusters (clusters 8–23) showed moderate but significant elevations in risk, often restricted to one or a few municipalities and lasting only a single week.

### Short‑duration and point clusters

Short‑duration and point clusters were also detected. An example occurred in northern Mexico during week 4 (cluster 7), where six neighboring municipalities reported 31 cases despite an expectation of 0.30 (RR 104.8). A second short‑duration cluster emerged in central Mexico (cluster 11) during weeks 47–49, comprising 80 cases versus 21.2 expected (RR 3.8). Several single‑municipality point clusters (clusters 9, 15, 16, and 18) reported 5–21 cases over brief intervals, with RR values exceeding 50.

### Low‑intensity widespread cluster

Cluster 10 was a large, diffuse aggregation spanning 244 municipalities across central and northern Mexico during weeks 42–43. It contained 157 observed cases versus 60.3 expected (RR 2.64).

### Clusters with borderline significance

Two clusters met reporting criteria despite higher p‑values. Cluster 20 (RR 32.2) comprised six cases during weeks 36–37 (p = 0.114), while cluster 21 (RR 74.8) involved four cases in week 10 (p = 0.410). These clusters were retained under the no‑geographical‑overlap rule. Table 1 summarizes the significant space–time clusters of measles detected using a discrete Poisson model.

**Table 1:** Space–time clusters of measles detected using a discrete Poisson model, Mexico, 2025–26.

Cluster	Municipalities (*n*)	Time frame (weeks)	Population	Cases (n)		RR	Notes
				Observed	Expected		
1	89	6–30	2,438,590	4,012	75.0	103.7	Largest, highest-intensity regional cluster (north)
2	113	45–49	9,346,839	1,493	57.5	31.5	Late-period central–western cluster
3	9	32–41	383,520	295	4.7	64.8	High-intensity mid-period cluster (Bajío)
4	1	25–49	18,776	134	0.6	235.8	Extreme point cluster (Oaxaca)
5	22	46–50	1,515,892	193	9.3	21.2	Short-duration Chiapas cluster
6	35	32–39	733,741	108	7.2	15.1	Mid-period northern cluster
7	6	Week 4	241,164	31	0.3	104.8	Explosive early-period northern cluster
8	7	48–49	2,193,664	58	5.4	10.8	Puebla–Tlaxcala peri-urban cluster
9	1	40–42	105,242	21	0.4	54.2	High-risk point cluster (Morelos)
10	244	42–43	24,480,477	157	60.3	2.6	Large, diffuse, low-intensity national corridor
11	8	47–49	5,741,841	80	21.2	3.8	Mexico City peri-urban cluster
12	8	Week 43	522,722	19	0.6	29.6	Short, intense Chiapas cluster
13	3	37–40	67,096	13	0.3	39.4	Puebla–Morelos micro-cluster
14	5	10–14	147,996	17	0.9	18.7	Early-period Zacatecas–Durango cluster
15	1	14–18	93,018	14	0.6	24.5	High-risk point cluster (Michoacán)
16	1	38–41	17,652	8	0.09	92.1	Very high-risk point cluster (Puebla).
17	1	22–24	43,455	7	0.16	43.7	Yucatán point cluster
18	1	10–17	4,127	5	0.04	123.1	Extreme-risk micro-cluster (Tamaulipas)
19	1	32–36	9,783	5	0.06	83.1	High-risk point cluster (Zacatecas)

The estimates were generated using a retrospective space–time analysis in SaTScan v10.3.3 with a discrete Poisson model. The scan employed cylindrical windows with variable spatial radii (up to 50% of the population at risk) and temporal durations ranging from 1 to 25 weeks (50% of the study period). Expected cases were calculated under the assumption of constant risk across space and time, proportional to municipal population. The relative risk represents the ratio of incidence within the cluster to that outside it. Statistical significance was assessed through 999 Monte Carlo replications, with p values derived from comparing the observed log‑likelihood ratio with the simulated distribution. Only non‑overlapping clusters were reported according to SaTScan criteria.

## Discussion

The 2025–26 measles resurgence in Mexico unfolded as a highly heterogeneous epidemic, with marked subnational variation in reported incidence across municipalities. Using municipal‑level surveillance data and Kulldorff’s space–time scan statistic, we identified 23 clusters of elevated risk, 19 of which were statistically significant. A dominant, long‑duration cluster in northern Mexico accounted for nearly half of all confirmed cases, with a broad, contiguous geographic extent. Secondary clusters in western and central Mexico, along with brief high‑intensity events, occurred in multiple municipalities and time periods. A late‑phase, low‑intensity cluster spanning 244 municipalities occurred during weeks 42–43. Taken together, these findings indicate that the resurgence comprised a combination of prolonged regional clusters and brief high‑intensity events, rather than a uniform pattern of spread across the country.

The spatially focal nature of the resurgence aligns with global reports documenting post‑pandemic measles resurgences in settings where routine immunization declined and surveillance systems were strained [29,30]. Previous analyses of measles in Mexico have emphasized the country’s long period of elimination and the importance of maintaining high coverage to prevent re‑establishment of endemic transmission [31]. However, no prior study has systematically characterized the geographic footprint or temporal evolution of the 2025–26 measles resurgence in Mexico.

The identification of large, contiguous clusters in northern and western Mexico is consistent with earlier work showing pronounced geographic variation in vaccination coverage and persistent pockets of under-immunized populations [32]. Available evidence suggests that several states within the primary northern cluster (Chihuahua, Durango, Sonora) experienced first-dose MCV coverage below 90% during 2019–2023 [33]. National MCV1 coverage declined from 88% in 2019 to 84% in 2020 during the COVID-19 pandemic, recovering partially to 91% by 2023 [15], but persistent subnational gaps remained well below the 95% herd immunity threshold required to prevent measles transmission [34]. Analysis of National Health and Nutrition Survey (ENSANUT) data revealed that MMR non-vaccination increased from 10.2% in 2012 to 29.1% in 2021 nationally, with state-level variation ranging from 13.1% to 72.5% [33]. These documented gaps created the epidemiological conditions necessary for sustained regional transmission following viral introduction.

The earliest confirmed cases occurred in unvaccinated Mennonite communities in Chihuahua, where an estimated 40,000 individuals reside with historically low vaccination acceptance. The later geographic expansion and prolonged transmission period show that cases subsequently appeared across a wider set of municipalities with differing vaccination coverage levels. The detection of extreme microclusters parallels observations from other countries where small, highly susceptible communities have generated intense but short-lived outbreaks [35,36]. This study adds to the literature by providing the first national, municipality-level spatiotemporal characterization of the resurgence, revealing patterns that were not visible in aggregate national reports.

These vulnerability patterns reflect systematic disparities affecting specific population subgroups. Analysis of the 2025 measles epidemic across Mexico demonstrated that indigenous individuals, while comprising 29.1% of confirmed cases nationally, accounted for 76% of all deaths, with complication rates significantly higher among indigenous patients (50.3% vs. 41.6%; p = 0.033) [37]. Historical vaccination data reveal that indigenous populations have MMR coverage up to 30 percentage points lower than non-indigenous populations [38], disparities that are compounded by geographic isolation, limited healthcare infrastructure in rural areas, institutional distrust, and logistical barriers including inadequate cold-chain storage and insufficient trained personnel in hard-to-reach communities [39]. In Chihuahua, the epicenter of sustained transmission identified in our analysis, viral spread extended beyond Mennonite communities to affect indigenous groups in the Sierra Tarahumara and seasonal agricultural workers with compromised vaccination access during harvest-season mobility [40]. The convergence of very high marginalization, rural residence, and indigenous status in high‑risk municipalities shows that cases occurred across multiple underserved populations rather than being confined to isolated communities.

Several factors have been proposed in previous studies to contextualize clustering patterns. The magnitude and duration of the primary northern cluster suggest that immunity gaps had accumulated to a level sufficient to sustain prolonged chains of transmission, facilitated by high mobility and interconnected municipal networks, particularly among young adult cohorts born during the 1-to-2-dose transition period [41,42]. Declines in routine childhood vaccination since 2018 and COVID‑19–related service disruptions have been described in prior literature as conditions that can increase population‑level susceptibility to measles outbreaks [43,44].

These high‑intensity, short‑duration events occurred in small populations and have been described in previous studies as consistent with settings where low immunity and delayed detection can coincide with rapid household or community‑level spread [32]. Their short duration resembles patterns described in previous studies in which measles transmission declined rapidly once the number of susceptible individuals within tightly connected communities became limited [35]. The late‑phase diffuse cluster occurred after earlier waves and is consistent with patterns reported in previous studies where broader geographic dispersion has been documented during later stages of measles outbreaks, including periods of increased mobility or shifts toward urban areas [36]. Together, these patterns align with prior studies describing how structural vulnerabilities, mobility, and surveillance performance can coincide in shaping the observed distribution of measles cases.

### Strengths and limitations

A major strength of this study is the use of high‑resolution municipal data and a robust, widely validated statistical framework to detect and quantify spatiotemporal clusters. The analysis captures both sustained regional transmission and short‑duration microoutbreaks, offering a granular view of epidemic dynamics. The use of open‑access surveillance data and reproducible SaTScan inputs enhances transparency and replicability.

However, several limitations warrant consideration. First, reliance on diagnosis date rather than symptom onset may introduce temporal imprecision, although this limitation is inherent to the public dataset. This imprecision may affect the exact timing of cluster initiation and termination, particularly for short‑duration events. Second, a reporting gap in late 2025 required temporal realignment to maintain sequence continuity for scan statistics. While this approach preserved the analytical framework, it introduces several potential biases. Any cases occurring during the unreported period (approximately mid-December 2025) would not be captured in our analysis, potentially underestimating the true magnitude of transmission during the Decline-to-Late phase transition. Clusters with temporal windows spanning this period may have artificially truncated durations or underestimated case counts, and if transmission intensity varied significantly during the gap, our characterization of the late-phase diffuse cluster (cluster 10, weeks 42–43) and the temporal boundary between outbreak phases may be affected. These uncertainties may influence both the apparent size of late‑phase clusters and the precision with which phase transitions are delineated. Additionally, state-level heterogeneity in reporting completeness during this period could have differentially impacted the detection of secondary clusters outside Chihuahua, where surveillance infrastructure may have been more strained. As a result, regional comparisons of cluster magnitude or persistence should be interpreted with caution. These factors should be considered when interpreting cluster duration, magnitude, and the precise timing of epidemic phase transitions. Third, under‑ascertainment of cases, particularly in municipalities with limited surveillance capacity, may have led to underestimation of cluster magnitude or failure to detect smaller clusters in remote areas. This could bias the spatial distribution of detected clusters toward better‑resourced municipalities. Fourth, the scan statistic assumes Poisson distribution proportional to population size, which may not fully capture heterogeneities in contact patterns or health‑seeking behavior. Moreover, SaTScan results are sensitive to parameter selection (e.g., maximum spatial and temporal window sizes), and different specifications could yield variations in cluster detection.

Fifth, no formal assessment of global spatial autocorrelation was performed; the spatial heterogeneity described in this study is based on the scan statistic and visual patterns rather than metrics such as Moran’s I. It is important to note that spatial heterogeneity was not formally assessed using global measures; instead, the patterns described here reflect localized excess risk identified through the scan statistic and visual inspection of municipal‑level incidence. The use of municipal centroids as proxies for population distribution and the absence of adjustments for temporal trends or global spatial dependence may influence the detection, size, and duration of clusters.

Accordingly, findings reflect localized clustering of excess risk rather than a characterization of broader spatial dependence across the municipal distribution of incidence. Sixth, the analysis does not incorporate vaccination coverage or mobility data at the individual level, which could further elucidate the drivers of cluster formation and transmission dynamics. The absence of these data limits the ability to contextualize clusters in terms of underlying susceptibility or movement patterns, and therefore the findings should be interpreted as descriptive rather than explanatory.

### Implications for policy and practice

The marked contrast between sustained regional transmission and localized surges underscores the need for a dual‑track public health strategy. First, targeted “mop‑up” immunization campaigns should prioritize the high‑risk northern and western regions identified in this study, where sustained transmission was most pronounced. Strengthening routine immunization in these areas is essential to prevent reestablishment of endemic circulation. Second, the detection of high‑intensity microclusters highlights the need for rapid‑response capacity at the municipal level. Surveillance systems must be capable of detecting small increases in incidence and deploying interventions within days to prevent localized introductions from evolving into regional outbreaks. Third, the late‑phase diffuse cluster suggests that national‑level efforts to restore routine vaccination, rebuild trust in immunization programs, and improve cross‑jurisdictional coordination remain critical. Given the descriptive nature of this analysis, these considerations should be interpreted as contextual insights rather than prescriptive recommendations. Integrating spatiotemporal analytics into routine surveillance could support more agile, geographically tailored responses. Such applications should be viewed as potential areas for operational strengthening rather than direct policy directives derived from causal inference.

## Conclusions

The 2025–26 measles resurgence in Mexico exhibited marked subnational heterogeneity, with both sustained regional transmission and multiple short‑duration microoutbreaks. Large, contiguous clusters in northern and western Mexico, together with high‑risk point clusters, demonstrate that national‑level indicators can obscure substantial local vulnerability. While this study is descriptive, the identification of where and when excess incidence occurred provides actionable context for strengthening subnational surveillance and restoring routine immunization. Spatiotemporal analytic tools can complement these efforts by revealing localized patterns of excess risk and supporting more geographically tailored public health strategies.

## Supporting information

S1 Fig Geographic reference map of Mexico’s 32 federal entities. This map shows the political boundaries of Mexico’s states, which provide the administrative context for the municipality‑level measles surveillance data analyzed in this study. The figure serves as a geographic reference to help readers locate regions mentioned in the results and interpret the spatial extent of identified clusters.
(TIF)
